# Psychiatric and Behavioural Side Effects Associated With Perampanel in Patients With Temporal Lobe Epilepsy. A Real-World Experience

**DOI:** 10.3389/fneur.2022.839985

**Published:** 2022-03-07

**Authors:** Anna Mammì, Edoardo Ferlazzo, Sara Gasparini, Valentina Bova, Sabrina Neri, Angelo Labate, Giovanni Mastroianni, Concetta Lo Bianco, Vittoria Cianci, Umberto Aguglia

**Affiliations:** ^1^Department of Medical and Surgical Sciences, Magna Graecia University, Catanzaro, Italy; ^2^Regional Epilepsy Centre, Great Metropolitan Hospital BMM, Reggio Calabria, Italy; ^3^Neurology Unit, Department of BIOMORF, University of Messina, Messina, Italy

**Keywords:** adverse effects, focal epilepsy, psychosis, irritability, anti-seizure medications

## Abstract

Psychiatric and behavioural side effects are common, undesirable effects associated with antiseizure medication use. Temporal lobe epilepsy is the most common focal epilepsy in adults and it is frequently associated with drug resistance. Patients with intractable epilepsy are more likely to have psychiatric and behavioural side effects when taking antiseizure medications and seem to be at higher risk for psychiatric comorbidities. Perampanel is a novel anti-seizure medication approved for focal and generalised epilepsies as add-on therapy. This is a 12-week short-term observational prospective study on people with focal epilepsy consecutively recruited from an Italian tertiary epilepsy centre, aimed to compare incidence and severity of psychiatric and behavioural side effects associated with perampanel use in patients with temporal lobe epilepsy as compared to other focal epilepsies. All patients received add-on perampanel according to indication and clinical judgement. Incidence and severity of psychiatric and behavioural side effects were rated by Neuropsychiatric Inventory Questionnaire. All patients enrolled answered the questionnaire before starting perampanel and after 12 weeks of treatment. We found no significant difference in terms of incidence and severity of psychiatric and behavioural side effects associated with perampanel in patients with temporal lobe epilepsy as compared to other focal epilepsies. In line with the literature, the most common adverse effects were “irritability” for both groups and “aggression” for patients with other focal epilepsies.

## Introduction

Psychiatric disturbances are more common in people with epilepsy than in the general population (1). People with epilepsy (PWE) may experience psychiatric and behavioural side effects (PBSEs) related to antiseizure medications (ASMSs), including depressed mood, anxiety, psychosis, obsession, personality traits, irritability, aggressive behaviour, and suicide (2, 3).

People with drug-resistant epilepsy (seizures failing to disappear with two or more ASMs) are more likely to have PBSEs when taking ASMs (4). Furthermore, pre-existing psychiatric history is a strong predictor of PBSEs in adult patients with epilepsy (5). Temporal lobe epilepsy (TLE) is the most common type of focal epilepsy and it is often associated with drug resistance (6). In relation to psychiatric disturbances, a meta-analysis demonstrated that the estimated prevalence of psychosis was higher among individuals with TLE, implicating that the temporal lobe area may be a key brain region, sharing the pathophysiology of TLE and psychiatric disorders (7). Perampanel (PER) is licenced as an add-on treatment for focal onset seizures starting from age 4 and for generalised-onset tonic-clonic seizures in idiopathic generalised epilepsies in people aged 7 years and older (8). PER is a selective, non-competitive antagonist of α-amino-3-hydroxy-5-methyl-4-isoxazolepropionic acid (AMPA)-type glutamate receptors on post-synaptic neurons and it is the first-in-class AMPA receptor antagonist which acts by reducing glutamatergic transmission (9–11).

Perampanel (PER) treatment is generally well tolerated, with dizziness, somnolence, headache, and fatigue being the most common treatment-emergent side effects (12–14). Pooled data from phase III studies showed elevated rates of PBSEs, in particular hostility/aggression among patients receiving PER compared to those receiving placebo (15, 16). It is therefore reasonable to hypothesise that PER may cause a higher incidence of PBSEs in people with TLE, who are more prone to psychiatric comorbidities and ASMs side effects (17). Here we aim to investigate the prevalence and severity of PBSEs associated with PER use as add-on therapy in patients with TLE as compared to other focal epilepsies.

## Materials and Methods

The present prospective observational study involved adults with focal epilepsy recruited from the Italian tertiary Centre for epilepsy care (Regional Epilepsy Centre, Grande Ospedale Metropolitano “Bianchi-Melacrino-Morelli”, Reggio Calabria, Italy). Inclusion criteria were the following: (a) age >18 years; (b) inadequate response to one or more ASMs; (c) diagnosis of focal epilepsy according to International League Against Epilepsy (ILAE) (18, 19); (d) no change in the ASMs (neither ASM introduction nor changes of dose of pre-existent ASM) during the previous 30 days. Exclusion criteria were: (a) generalised epilepsy; (b) past or current history of alcohol or drug abuse; (c) contraindications to PER use. All recruited participants were started on a once-daily add-on PER at bedtime. An initial dose of 2 mg/day was administered and subsequently increased by 2 mg every 2/4 weeks based on clinical judgement up to a maximum dose of 12 mg/day.

### Procedure

The following data have been collected at baseline for each patient: age, gender, epilepsy aetiology, epileptic syndrome, pre-existing psychiatric comorbidities (i.e., depression, anxiety, personality disorder, psychosis, and psychogenic non-epileptic seizures), ASMs used prior to PER, concomitant ASMs, and duration of PER treatment. The diagnosis of focal epilepsy was made based on clinical, EEG, and MRI criteria (18). Patients were divided into two groups: group 1 (TLE) and group 2 (other focal epilepsies). Lobar localisation was established according to the combination of seizure semiology, ictal/interictal EEG findings, and imaging results based on ILAE classification (19). Incidence and severity of PBSEs in both groups were rated with Neuropsychiatric Inventory-Q (NPI-Q; see below) (20) that was administered to the participants' caregivers before starting PER and after 12 weeks of treatment. PBSEs were searched in all subjects who received at least one dosage of PER and had at least one follow-up clinical evaluation. PBSEs were considered as associated with PER if they were absent before PER or were already present before PER but clearly worsened (score after PER ≥ 1) after PER introduction. Suicidality was also evaluated at each visit. All patients that received PER were included in the analysis.

### NPI-Q

Incidence and severity of PBSEs were assessed through NPI-Q scores at baseline and follow-up times. NPI-Q is a Brief Clinical Form of the Neuropsychiatric Inventory, covering 12 neuropsychiatric symptom domains: depressed mood, euphoria, anxiety, apathy, irritability, aberrant motor behaviours, disinhibition, night-time behavioural disturbances, delusions, hallucinations, appetite/eating disturbances, and aggression.

Furthermore, twelve-symptom domains are assessed by a written screening question that includes core symptom manifestations. Patients are asked to circle “yes” or “no” in response to each question, and to rate the symptoms present in the last 12 weeks if the answer is “yes”. Severity neuropsychiatric symptoms are assessed on a three-point scale (1-mild, 2-moderate, and 3-severe). The total NPI-Q severity score represents the sum of individual symptoms scores and ranges from 0 to 36.

### Statistical Analysis

Descriptive data are expressed as mean ± standard deviation or number (percentage), as appropriate. Fisher's exact test was used to compare proportions for dichotomic variables in both groups. The nonparametric Mann–Whitney–Wilcoxon (MWW) rank-sum test was used to compare the severity of symptoms. A *p* < 0.05 was considered statistically significant. All tests were two-tailed. Jamovi 2.2.2 (The Jamovi Project, 2021) (21) was used for all statistical procedures.

## Results

### Participants

Demographic and clinical features of all subjects are shown in Table 1. We enrolled 66 participants (37 women; mean age: 46.9 ± 16.6 years). Among the participants, 39 (59.1%) had TLE (21 women; mean age: 50.3 ± 16.9), whereas 27 (40.9%) had other focal epilepsies (16 women; mean age: 42.9 ± 13.6), including nine with frontal lobe epilepsy, nine with parietal lobe epilepsy, four with occipital lobe epilepsy, and five with multifocal or unknown localisation. Before PER introduction, 28 subjects (42.4%) had psychiatric comorbidities.

**Table 1:** Demographical and clinical features as a function of focal epilepsies.

	**Group 1 (TLE) (*n =* 39)**	**Group 2 (Other focal epilepsies) (*n =* 27)**	***p* value**
			
N (%)	39 (59.1)	27 (40.9)	0.17
Age, years (mean ± SD)	50.3 ± 16.9	42.9 ± 13.6	0.06
Sex M/F, n (%)	18/21 (46.2/53.8)	11/16 (40.7/59.3)	0.80
Previous ASMs			
1–3, n (%)	24 (61.5)	12 (44.4)	0.17
4–5, n (%)	5 (12.8)	7 (25.9)	0.17
≥6, n (%)	10 (25.6)	8 (29.6)	0.72
PER dose, mg/day (mean ± SD)	7.2 ± 2.7	7,1 ± 2.5	0.81
Concomitant ASMs (mean ± SD)	2.1 ± 0.7	2.1 ± 0.7	0.96
1, n (%)	8 (20.5)	5 (18.5)	0.84
2, n (%)	20 (51.3)	15 (55.6)	0.73
3, n (%)	10 (25.6)	6 (22.2)	0.75
4, n (%)	1 (2.6)	1 (3.7)	0.79
Psychiatric disorders (PD) n (%)	19 (48.7)	9 (33.3)	0.31
Depressed mood n (%)	14 (35.9)	5 (18.5)	0.17
Anxiety n (%)	4 (10.3)	3 (11.1)	1
Psychosis n (%)	7 (17.9)	4 (14.8)	1
Concomitant psychotropic drugs n (%)	19 (48.7)	9 (33.3)	0.31
Antidepressant n (%)	14 (35.8)	5 (19.5)	0.79
Anxiolytic n (%)	16 (41)	8 (29.6)	0.43
Antipsychotic n (%)	7 (17.9)	4 (14.8)	1

At the time of starting PER, participants took an average of two ASMs. All patients were drug-resistant according to Kwan et al. (22). All the patients received a starting dose of PER of 2 mg/day, with an increase of 2 mg/day every 2–4 weeks when indicated, up to a maximum dose of 12 mg/day.

At the time of follow-up, after 12 weeks, 59 (89.4%) patients were still taking PER, whereas seven patients (10.6%) discontinued PER, two (3%) because of PBSEs, five (7.6%) because of other adverse effects (dizziness, somnolence, and asthenia). Among the patients who discontinued PER, 6/7 had TLE and 1/7 extra-TLE (*p* = 0.23). PER median dose after 12 week-treatment was 7.2 ± 2.6 mg/day for all patients. No significant differences were found between groups 1 and 2 in terms of demographic variables.

### Incidence and Severity of PBSEs

Incidence and severity of PBSEs related to PER were rated by NPI-Q patients scores signed at baseline and follow-up.

Considering the whole sample, 13/66 patients (19.7%) experienced from 1 to 3 PBSEs, 13/66 (19.7%) experienced “irritability”, 2/66 (3%) experienced “aggression”, 1/66 (1.5%) had “depression”, 1/66 (1.5%) had “aberrant motor behaviours”, and 1/66 (1.5%) had “night-time behavioural disturbances”.

In group 1, 8/39 (20.5%) subjects experienced from 1 to 3 PBSEs, 1/39 (2.6%) experienced “depression”, 8/39 patients (20.5%) experienced “irritability”, 1/39 (2.6%) had “aberrant motor behaviours”, and 1/39 (2.6%) had “night-time behavioural disturbances”. The median severity score was 5.5 (IQR, 3–7,25). In group 2, 5 subjects experienced PBSEs, all patients (18.5%) had “irritability” with two of them experiencing aggression too. No difference was found between the two groups (for more details see Table 2). Suicide attempts were not experienced by any patients.

**Table 2:** Incidence and severity of BPSEs as functions of focal epilepsies.

	**Group 1 (TLE) (*n =* 39)**	**Group 2 (Other focal epilepsies) (*n =* 27)**	***p* value**
Patients experienced PBSEs n (%)	8 (20.5)	5 (18.5)	0.95
The severity of symptoms median (IQR)	5.5 (IQR, 3–7.25)	5 (IQR, 3–7)	0.82
Depressed mood n (%)	1 (2.6)	0	0.40
Irritability n (%)	8 (20.5)	5 (18.5)	0.84
Aberrant motor behaviours n (%)	1 (2.6)	0	0.40
Night-time behavioural disturbances n (%)	1 (2.6)	0	0.40
Aggression n (%)	0	2 (7.4)	0.08

## Discussion

In the present study, we found no significant difference in terms of incidence and severity of psychiatric and behavioural side effects associated with PER in people with TLE as compared to other focal epilepsies. PBSEs appeared in 19.7% of patients, in agreement with literature data, without significant difference between people with TLE (20.5%) and other focal epilepsies (18.5%). Indeed, double-blind randomised controlled trial (RCT) studies showed that PER is generally well tolerated with the most common adverse events being dizziness, somnolence, headache, fatigue, irritability, and aggression in about 20% of patients (12–14). Moreover, several real-world studies showed a similar rate of PBSEs at 1 to 3-year follow-up (23–27). Mild to moderate PBSEs (mainly irritability, anxiety, verbal aggressivity, agitation/restlessness, depression, psychosis, and suicidal ideation) were reported in about 15–25 % of patients. Furthermore, people with a history of psychiatric comorbidities showed a significantly higher risk of developing psychosis and suicidal ideation.

To our knowledge, no other study comparing the incidence and severity of PBSEs associated with PER use in PWE and different lobar localisation has been conducted so far. Likewise, there aren't any studies exploring the incidence of PBSEs associated with other ASMs in different types of focal epilepsies, although it is reported that PBSEs are more likely to occur in people with psychiatric comorbidity and who are considered to be at risk of psychiatric disorders, like TLE people (28).

In our study, we found no higher incidence of PBSEs in people with TLE. We might hypothesise that concomitant ASM and other psychotropic medications are taken may have mitigated psychiatric adverse side effects. However, we found no difference in terms of concomitant medications in our two groups and this might be explained by the small sample size of the present study.

Moreover, people with TLE may have an increased risk of developing psychiatric comorbidity without a higher risk to experience PBSEs associated with ASMs because of the different pathogenic mechanisms underlying both psychiatric symptoms.

It is well known that a strong and bidirectional relationship between epilepsy and psychiatric disturbance. Hence, it could be hypothesised a common etiopathology of neurological and psychiatric disorders based on both genetic and environmental factors. On the other hand, PBSEs associated with ASMs are pharmacologically induced phenomena and are strictly dependent on ASM mechanisms of action, interactions with other psychotropic medications, pre-existent psychiatric disturbances, personality and life quality of PWE, change in seizure frequency (force normalisation), and other personal risk and protective factors of psychiatric symptoms.

Our study has some limitations. It is an observational study in which participants are not randomised and a control group of patients treated with other ASMs is not included. Furthermore, the sample size is limited and follow-up time (12 weeks) may be too short to exclude the occurrence of PBSEs. So, our findings require further validation from studies with larger sample sizes and longer follow-up.

## Data Availability Statement

The original contributions presented in the study are included in the article/supplementary material, further inquiries can be directed to the corresponding author.

## Ethics Statement

Ethical review and approval was not required for the study on human participants in accordance with the local legislation and institutional requirements. Written informed consent for participation was not required for this study in accordance with the national legislation and the institutional requirements.

## Author Contributions

VC and AL contributed to conception and design of the study. GM, VB, and SN organised the database. AM and CB wrote the first draft of the manuscript. EF and SG wrote sections of the manuscript. UA contributed to manuscript revision. All authors contributed to manuscript revision, read, and approved the submitted version.

## Conflict of Interest

The authors declare that the research was conducted in the absence of any commercial or financial relationships that could be construed as a potential conflict of interest.

## Publisher's Note

All claims expressed in this article are solely those of the authors and do not necessarily represent those of their affiliated organizations, or those of the publisher, the editors and the reviewers. Any product that may be evaluated in this article, or claim that may be made by its manufacturer, is not guaranteed or endorsed by the publisher.
